# Intracranial Angioplasty via Type II Proatlantal Intersegmental Artery

**DOI:** 10.7759/cureus.47724

**Published:** 2023-10-26

**Authors:** Denis Babici, Phillip M Johansen, Nadia Sial, Brian Snelling

**Affiliations:** 1 Neurology, Florida Atlantic University Charles E. Schmidt College of Medicine, Boca Raton, USA; 2 Neurological Surgery, Florida Atlantic University Charles E. Schmidt College of Medicine, Boca Raton, USA; 3 Neurosurgery, Boca Raton Regional Hospital, Boca Raton, USA

**Keywords:** catheter angiogram, vertebrobasilar stroke, cta, angioplasty, pia type ii

## Abstract

A proatlantal intersegmental artery (PIA) is an exceedingly rare primitive anastomosis between the carotid and vertebrobasilar circulations. PIAs may be accompanied by ipsilateral or bilateral vertebral artery (VA) agenesis and can originate from the cervical internal carotid artery (ICA, type I) or external carotid artery (ECA, type II) before eventually joining the vertebrobasilar system. Several authors have described this anomaly in different clinical scenarios, but to our knowledge, there are no studies documenting VA angioplasty through a type II PIA in the setting of vertebrobasilar stroke. We present the case of vertebrobasilar stroke in which the right VA did not originate from the right subclavian artery but instead from the ECA. The patient was subsequently determined to have a type II PIA. We performed right VA angioplasty via the PIA, followed by partial restoration of vertebrobasilar blood flow. This is the first documented case of intracranial vertebral angioplasty through a type II PIA and serves as a reminder for neuroendovascular surgeons about persistent fetal circulation. In such instances, an angiogram of both the ICA and ECA should be performed to exclude right VA stenosis and visualize persistent fetal circulation. This case underscores the complexity of arterial thrombotic events, the beneficial role of endovascular intervention, and the necessity of future studies to identify the optimal treatment methods for vertebrobasilar stroke.

## Introduction

Approximately 25% of ischemic strokes are due to occlusion of large posterior circulation arteries, such as the vertebral, basilar, and posterior cerebral arteries. In basilar artery occlusion (BAO), the clinical presentation varies based on the severity of the occlusion. Mild occlusion may lead to dizziness, headache, and dysarthria. In comparison, a complete occlusion can cause quadriplegia and locked-in syndrome. BAO is a rare but urgent situation that accounts for around 1% of all strokes and 8% of acute vertebrobasilar ischemic events [1,2]. Advanced endovascular technology allows interventionalists to utilize novel techniques for basilar artery recanalization, especially when routine approaches are not possible.

A proatlantal intersegmental artery (PIA) is an example of a primitive carotid-vertebrobasilar anastomosis that exists transiently during fetal development. Persistence of such fetal circulation into adulthood is extremely rare and often discovered incidentally during an angiography [3]. There are two types of PIA. The type I variant arises from the internal carotid artery (ICA), while the type II variant most commonly originates from the external carotid artery (ECA). Both types of PIAs course upward before turning dorsally to rest on the superior aspect of the transverse process of C1. PIAs typically join the V3 segment of the vertebral artery (VA) but may also continue alongside the VA, passing through the foramen magnum before merging into the vertebrobasilar system. We present the case of right VA recanalization through a type II PIA for subsequent anterograde balloon angioplasty of the right intracranial VA, followed by partial restoration of vertebrobasilar blood flow. To our knowledge, this is the first documented case of VA angioplasty through a type II PIA in the setting of vertebrobasilar stroke.

## Case presentation

**Figure 1:**
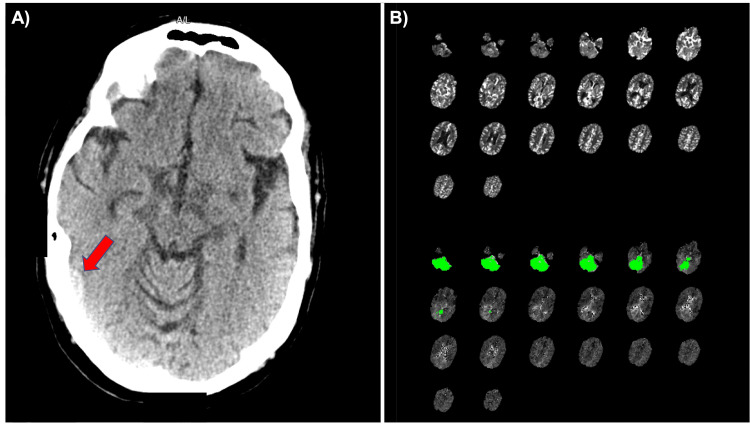
A. CT scan of the brain revealing a small right posterior parietal subdural hematoma. B. CT perfusion scan of the brain revealing ischemia of the cerebellar hemispheres bilaterally with small areas of core infarction and a moderate-to-large surrounding penumbra

**Figure 2:**
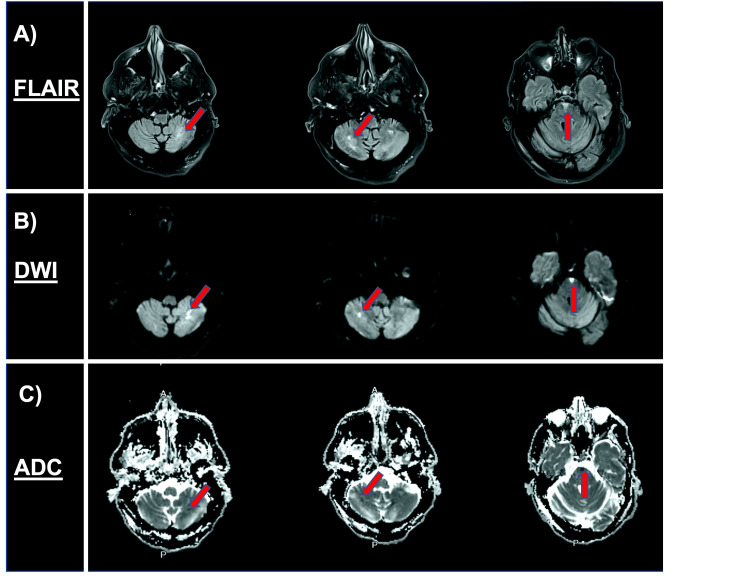
MRI of the brain: acute cerebellar and brainstem infarct FLAIR: fluid-attenuated inversion recovery; DWI: diffusion-weighted imaging; ADC: apparent diffusion coefficient

A 62-year-old male with a past medical history of a cerebrovascular event, syncope, type 2 diabetes mellitus, hypertension, hyperlipidemia, and multiple falls presented to the emergency department via emergency medical services due to slurred speech that began one hour prior to arrival. On admission, the patient was hemodynamically stable, and laboratory analysis was unremarkable. Physical examination was notable for severe dysarthria with intact comprehension and fluency as well as ocular bobbing with left lateral gaze palsy. Computed tomography (CT) of the head revealed a small right posterior parietal subdural hematoma (SDH) (Figure 1A), and a CT perfusion scan revealed ischemia of the cerebellar hemispheres bilaterally with small areas of core infarction and a moderate-to-large surrounding penumbra (Figure 1B). Magnetic resonance imaging (MRI) of the brain was positive for bilateral acute ischemic strokes in the cerebellum and pons (Figure 2). 

**Figure 3:**
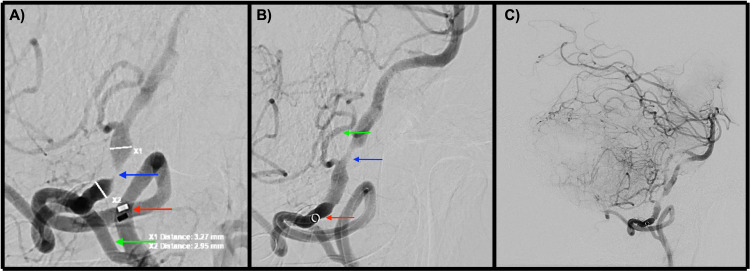
Catheter angiogram. A. Magnified lateral angiogram before angioplasty: red arrow: 5F intermediate catheter within the type II PIA; blue arrow: critical stenosis; and green arrow: occipital artery. B. Magnified lateral angiogram after angioplasty: red arrow: 5F intermediate catheter within the type II PIA; blue arrow: residual stenosis; and green arrow: right posterior inferior cerebellar artery. C. Lateral angiogram demonstrating improved flow to posterior circulation

Due to a recent fall with subsequent subacute subdural hemorrhage, the patient was not a candidate for thrombolysis. The neurosurgeon evaluated the patient for hematoma evacuation, but given the small size, they determined that the patient was not a candidate for craniotomy either. At this time, we opted for a CT angiogram of the head and neck, which revealed high-grade stenosis of the right intracranial VA, and the left VA P1, V2, and V3 segments were narrow in caliber. The proximal left V4 segment was occluded with distal reconstitution (Figure 2). The neuroendovascular surgeon was consulted, and the decision was made to proceed with a cerebral angiogram with possible balloon angioplasty of the right VA. A catheter angiogram of the ICA and ECA was performed, during which the physician observed that the patient's VA did not originate from the right subclavian artery but instead from the ECA. The patient was subsequently determined to have a type II PIA with critical stenosis (Figure 3A). The decision was made to proceed with the right VA angioplasty via his type II PIA. Moderate stenosis was seen following sub-maximal angioplasty (Figure 3B). The decision was made not to pursue intracranial stenting given the need for antiplatelet therapy in the setting of SDH. The procedure went without complications with partial restoration of vertebrobasilar blood flow (Figure 3C). On postoperative day 5, the patient reported the resolution of his symptoms and was subsequently discharged.

## Discussion

Communication of four separate fetal carotid-vertebrobasilar anastomoses plays an essential role in supplying the posterior circulation of the developing embryo. Such circulatory anastomoses begin to form when the embryo is only 4-5 mm in size and disappear when the embryo reaches 7-12 mm in size [4-6]. The first anastomosis involves the trigeminal artery, followed by the otic, hypoglossal, and proatlantal intersegmental arteries. Failure of these communicating anastomoses to involute contributes to the persistence of such vascular anomalies in adults [6]. 

**Figure 4:**
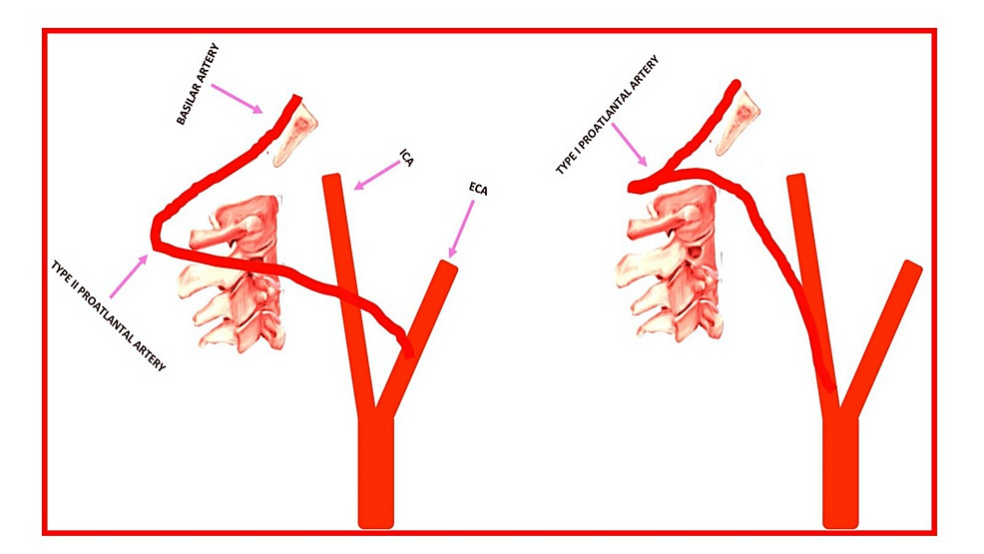
Types of proatlantal intersegmental artery: type I, which originates from the ICA and extends to the occipito‐C1 vertebral space and perforates the dura to join the intradural segment of the vertebral artery, and type II, which originates from the ECA and extends to the C1‐C2 vertebra and perforates the dura to join the intradural segment of the vertebral artery ECA: external carotid artery; ICA: internal carotid artery

There are two variants of persistent PIA, both of which are anomalies if observed in adulthood (Figure 4). PIAs may arise from the common, external, or internal carotid arteries and typically do so between C2 and C4. The type I variant arises from the ICA, while the type II variant originates from the ECA prior to joining the vertebrobasilar system [7]. Such features can help distinguish PIAs from other persistent fetal anastomoses, such as a hypoglossal artery, which originates from the ICA between C1 and C3 and enters the hypoglossal canal prior to joining the vertebrobasilar system. As in our case, persistent fetal anastomoses are commonly associated with other vascular anomalies, such as VA aplasia or hypoplasia. Although patients with PIAs are generally asymptomatic, they remain at increased risk of posterior circulation hemorrhage and ischemia secondary to altered hemodynamics as well as occlusion of these variant arteries [5]. 

Recanalization of large vessel occlusion through patent cerebral or cervical collateral vessels may be an option when direct access to the vertebrobasilar system is difficult or impossible. Data regarding the morbidity and mortality of BAO reveal that aggressive anterograde (and possibly retrograde) recanalization of the native posterior circulation is a feasible method in treating acute ischemic strokes of the basilar artery [2]. However, there is a paucity of literature describing PIAs in a clinical context. There is documented evidence of type I PIAs causing infarction of the rostral brainstem [8], paramedian pontine arteries (when associated with ICA stenosis) [9], watershed areas [10], posterior circulation [10], and middle cerebral artery (MCA, also accompanied by an ipsilateral trigeminal artery) [11]. Type II PIAs have been reported as causing transient hand weakness and amaurosis fugax (when associated with severe ICA stenosis) [12], as well as infarction of the MCA (when associated with ICA stenosis) [13]. Additionally, Ishiguro et al. suggested a steal phenomenon from the anterior to posterior circulation via a type II PIA, which resulted in infarction of the MCA [14]. Berger et al. described ICA stenosis, a type II PIA with tight stenosis at the origin, and bilateral VA agenesis which led to simultaneous hemispheric and vertebrobasilar ischemic cerebrovascular disease [3]. The four primitive carotid-vertebrobasilar anastomoses sometimes persist into the adult period. The persistent primitive trigeminal artery (PTA) is the most common persistent primitive carotid-vertebrobasilar anastomoses with an incidence of approximately 0.5-0.7% [15]. The PTA commonly originates from the intracavernous ICA. Less commonly, the PTA may arise from the petrous ICA. Another type is the persistent primitive otic artery (POA) that is a very rare vessel that has been reported just eight times in the literature [16]. The persistent primitive hypoglossal artery (PHA) is the second most common persistent carotid-vertebrobasilar anastomosis with an incidence ranging from 0.027% to 0.29% [17]. The PHA usually arises from the posterior side of the cervical ICA between the C1 and C3 levels and traverses the hypoglossal canal to form the vertebrobasilar artery [17].

However, there is no literature documenting VA angioplasty through type II PIA in the setting of a vertebrobasilar stroke. In the presented case, we struggled to determine the optimal mode of access required to perform the right VA angioplasty. Upon initial presentation, the patient's symptoms and imaging findings were suspicious of right VA occlusion. However, an angiogram revealed that his right VA originated from the right ECA rather than the subclavian artery. The patient was subsequently determined to have a type II PIA, and the only feasible option was to catheterize the right intracranial VA through the PIA and perform angioplasty. The result was a partial restoration of vertebrobasilar blood flow and improvement of the systems.

## Conclusions

The authors present a case of vertebrobasilar stroke in which the right VA did not originate from the right subclavian artery but instead from the ECA, with subsequent diagnosis of a type II PIA. The only option for management was to perform right VA angioplasty via the PIA. This is the first documented case of intracranial vertebral angioplasty through a type II PIA artery. In addition, this case serves as a reminder of the very rare cases of persistent fetal circulation and the importance of performing catheter angiogram of both the ICA and ECA. Evaluation of the ECA and subsequent observation of a PIA can completely change both the management and outcomes of the patient. This case underscores the complexity of arterial thrombotic events, the beneficial role of endovascular intervention, and the necessity of future studies to identify the optimal treatment methods for vertebrobasilar stroke.
